# P-880. State-wide Antibiogram using National Healthcare Safety Network Antimicrobial Resistance Option 2017–2023

**DOI:** 10.1093/ofid/ofaf695.1088

**Published:** 2026-01-11

**Authors:** Dipen Patel, Christopher D Evans, Melphine Harriott

**Affiliations:** HAI/AR, Tennessee Department of Health, Nashville, TN; Tennessee Department of Health, Nashville, Tennessee; TN Department of Health, Nashville, Tennessee

## Abstract

**Background:**

Antimicrobial resistance (AR) is a significant global health threat, leading to higher healthcare costs, longer hospital stays, and increased mortality rates. Antibiograms guide empiric therapy and strengthen antimicrobial stewardship programs (ASPs). However, many facilities lack facility-specific data, and facility-level antibiograms may not fully represent broader AR trends. To address this, we developed Tennessee’s first statewide antibiogram using data from the National Healthcare Safety Network (NHSN) AR Option.

**Methods:**

Organism isolates reported as susceptible, intermediate, resistant, non-susceptible, or not tested were extracted from the NHSN AR Option from 2017 to 2023. The percentage of susceptible isolates was calculated using only those tested for a specific drug, excluding intrinsically resistant organisms and those with < 30 isolates. The TN-ASP team, including an antimicrobial stewardship pharmacist, clinical microbiologist, and epidemiologist, finalized a publicly available interactive antibiogram using Tableau after a quality review to ensure accuracy.

**Results:**

The interactive dashboard lets users filter data by year, organism, onset, specimen, region, and antimicrobial class. From 2017–2023, 70 facilities reported 215,915 isolates, with *E. coli* being the most common organism of all isolates (44%). Urine was the most frequent specimen source (72%). The antibiogram analysis highlighted notable trends, such as *S. aureus* percent susceptibility remains below < 50% over the years (2017[47%], 2023 [46%]), while *K. pneumoniae* complex showed a decline in piperacillin/tazobactam susceptibility from 96% (2017) to 92% (2023).

**Conclusion:**

A statewide interactive antibiogram can assist healthcare providers and ASP teams, especially in rural areas where local AR data is often lacking, helping them make informed empirical therapy decisions. One limitation was that we could not verify the breakpoints used by facilities to determine susceptibility criteria. Future efforts will explore breakpoints and unusual susceptibilities. Despite the challenges, this statewide antibiogram provides valuable insights into statewide and regional AR trends, supports infection prevention efforts, and strengthens ASP initiatives.

**Disclosures:**

All Authors: No reported disclosures

